# Sedation options for the morbidly obese intensive care unit patient: a concise survey and an agenda for development

**DOI:** 10.1186/s40248-015-0007-2

**Published:** 2015-03-07

**Authors:** Riku Aantaa, Peter Tonner, Giorgio Conti, Dan Longrois, Jean Mantz, Jan P Mulier

**Affiliations:** Division of Perioperative Services, Intensive Care, Emergency Care and Pain Medicine, University of Turku, Turku, Finland; Department of Anaesthesiology and Intensive Care Medicine, Emergency Medicine Hospital Links der Weser GmbH, Bremen, Germany; Department of Intensive Care and Anaesthesiology, Università Cattolica del Sacro Cuore, Rome, Italy; Université Paris-Diderot, Hôpitaux Universitaires Paris Nord Val de Seine, Département d’Anesthésie Réanimation Chirurgicale, Hôpital Bichat-Claude Bernard, Paris, France; Anaesthesiology Department, Beaujon Hospital, AP-HP, Université Paris-Diderot, Paris, France; Department of Anaesthesiology, Intensive and Emergency Care, Sint Jan Brugge-Oostende, Ruddershove 10, Brugge, 8000 Belgium

**Keywords:** Clonidine, Dexmedetomidine, Intensive care, Ketamine, Obesity, Opioids, Propofol, Sedation, Volatile anaesthetics, Benzodiazepines

## Abstract

**Background:**

We offer some perspectives and commentary on the sedation of obese patients in the intensive care unit (ICU).

**Discussion:**

Sedation in morbidly obese patients should conform to the same broad principles now current in ICU practice. These include a general presumption against benzodiazepines as first-line agents. Opioids should be avoided in any situation where spontaneous breathing is required. Remifentanil is the preferred agent where continuous stable opioid levels using an infusion are required, because of its lack of context-sensitive accumulation. Volatile anaesthetics may be an option for the same reason but there are no substantial, controlled demonstrations of effectiveness/safety in short-term use in the ICU setting. Propofol is a valuable resource in the morbidly obese patients but the duration of continuous sedation should not exceed 6 days, in order to avoid propofol infusion syndrome. Alpha-2 agonists offer a range of theoretically positive features for the sedation of morbidly obese patients, but at present there is a lack of pharmacokinetic data and a critical mass of high-grade clinical data. Dexmedetomidine has the attraction of not causing respiratory depression or obstructive breathing during sedation and its sympatholytic effects should help deliver stable blood pressure and heart rate. Ketamine has a poor tolerability profile in adults so its use in the ICU context is largely confined to paediatrics.

**Conclusion:**

None of the agents currently available is ideal for every situation encountered in the management of morbidly obese patients. This article identifies additional research needed to place sedation practice of obese patients on a more systematic footing.

## Review

### Background

Nowadays obesity is undoubtedly more prevalent than in earlier times [1,2]. This change has filtered through the intensive care medicine, with the result that greater numbers of obese patients are encountered in critical care.

It is important to keep a sense of proportion about this trend. Firstly, it is our view that significant obesity-related challenges to the clinical management of critically ill patients emerge consistently only when body mass index (BMI) equals or exceeds 35 kg/m^2^. The proportion of critically ill patients meeting that condition is still relatively small (≈10%) [3]. Therefore, we consider it unhelpful to promote the idea that intensive care units (ICUs) throughout Europe are being tested beyond their capacities by large numbers of morbidly obese patients: this is not the case.

Moreover, many of the problems presented by morbidly obese patients in the perioperative period are well appreciated and the principles of good practice/risk reduction are established. These include a preference for the beach-chair position (and certainly the avoidance of supine positioning for intubation [4]), protocols and equipment specifications for difficult airway management [5], lung-protective ventilation [2] and the use of lung recruitment and adequate positive end-expiratory pressure (PEEP) [6]. Practical issues of staffing and the provision of suitable equipment (e.g. hoists and beds with suitable load-bearing capacity) must be acknowledged but not overstated, even though some of the solutions involve obvious economic costs.

Nevertheless, there are distinct clinical challenges in the management of morbidly obese patients. Notable among these are deleterious effects of obesity on the respiratory system, where the following difficulties may be encountered [7].

- Impaired function of respiratory muscles, diminished functional residual capacity and limitation of expiratory flow [8,9].
- Increased oxygen consumption, increased production of carbon dioxide and increased work of breathing [10].
- Increased upper airway resistance and propensity to obstructive sleep apnoea syndrome (OSAS) [8,11].
- Potential for obesity–hypoventilation syndrome, followed by pulmonary hypertension and right heart failure [12,13].

As a first step in any discussion about the challenges of managing the morbidly obese ICU patient it is important to appreciate that obesity is not merely a question of mass: qualitative factors of obesity have also to be considered. First, central or visceral obesity-more often encountered in men-appears to be a more malign form than peripheral obesity. Moreover, not all of the excess weight of morbidly obese patients is fat: lean body mass is also increased but usually not in proportion to adipose tissue. The fat:lean ratio is thus often different in obese patients than in otherwise comparable persons of normal weight. Distinctions between total body weight, lean body weight and ideal body weight are relevant to ventilator settings and drug dosage [14]. For example, the tidal volume calculated from the BMI will likely be far too large; correct (i.e. smaller) volumes need to be derived from the ideal body weight. Shifts in volumes of drug distribution in adipose tissue and body water, alterations in tissue/blood partition coefficients and clearance rates, changes in the affinity of drugs for plasma proteins and/or tissue components, increase in metabolization and altered regional/adipose blood flow may all be encountered in obese patients and affect the pharmacokinetic/pharmacodynamic profiles of sedative and anaesthetic drugs [15-17].

## Commentary

Use of sedatives in morbidly obese patients can be considered under several therapeutic categories/situations:

- non-invasive ventilation
- intubated ventilation
- short-term sedation (<24 h)
- long-term sedation (≥24 h).

There is overlap between several of these categories so that, for example, long-term sedation may be more likely in a setting of invasive mechanical ventilation.

### Sedatives used in the management of morbidly obese patients

We believe that sedation in morbidly obese patients should conform to the same broad principles now current in ICU practice. These include:

- maintaining the patient in a state of calm, awake cooperation
- multimodal sedation
- a strong presumption against first-line use of benzodiazepines and long-acting opioids.

Table 1 summarizes our views on the profiles of different sedatives as they relate to the management of morbidly obese patients. None of the agents currently available is ideal for every situation, though the reasons for saying so differ from one agent or drug class to another.

**Table 1:** **Effects of sedatives pertinent to their use/suitability in the management of morbidly obese patients**

**Sedative**		**Pharmacokinetic data or experience available in obese patients?**	**Non-intubated sedation**		**Intubated sedation**		**Short-term sedation**	**Long-term sedation**	**Deep sedation**
			**Upper airway patency**	**Respiratory drive**	**Respiratory rate**	**Muscle weakness**			
Alpha-2 agonists (primarily dexmedetomidine)		No	**+**	**+**	**+**	**+**	Yes	Yes	No
Benzodiazepines		No	--	**+**	-	-	No or maybe	**No**	Not alone
Propofol		Yes	-	-	-	-	**Yes**	Yes, but not to exceed 6 days without interruption	Yes
Volatile anaesthetics		Yes	**+**	**+**	**+**	**+**	**Yes**	?	Yes
				At ‘ambient’ (i.e. very low) concentrations					
S-ketamine		**?**	**+**	**+**	**+**	**+**	- or + (Assumes use at low dose, in combinations)	**Maybe yes,** maybe no	Yes
Opioids			-	--	- or **+**	**+**			No
	Remifentanil	Yes					Yes	Yes	
	Fentanyl, alfentanil, sufentanil	Yes					Yes	Yes	
	Morphine, oxycodone	Yes					Yes	Maybe yes, maybe no	
Barbiturates		Not resolved						In special groups	Yes

A plus sign indicates an affirmative effect or no adverse effect; a minus sign indicates at least a potentially adverse effect; two minus signs indicates a greater adverse effect or greater potential for an adverse effect.

**Bold type** indicates that there is at least some evidence to support this characterization. Assessments otherwise reflect expert opinion based on what the experts themselves regard as inadequate or incomplete objective evidence.

#### Benzodiazepines

The tide has been flowing against benzodiazepines as first-line sedatives for several years [18]. Various reasons may be adduced for this, including the possibility of a causal relation between prolonged benzodiazepine exposure and risk of delirium [18]. In any event, this general trend extends to obese patients and may be accentuated by the greater susceptibility of non-ventilated obese patients to compromised patency of the upper airways [19]. Effects on patency can be ameliorated by positioning manoeuvres [20] but remain a consideration. In common with other sedatives that act via γ-aminobutyric acid (GABA)-ergic pathways, benzodiazepines can also adversely affect respiratory drive and/or timing.

The availability of effective alternative agents now makes a strong case to favour non-benzodiazepine sedatives in mechanically ventilated patients [18,21]. Obesity does not detract from that case and may strengthen it.

#### Opioids

The propensity of opioids to cause or contribute to upper airway obstruction and respiratory depression makes a strong case against a reliance on these drugs in any situation where a spontaneous voluntary effort of breathing is required of the patient [22]. As a general precaution, several of us actively minimize the use of longer-acting opioids in morbidly obese patients.

The lack of context-sensitive accumulation of remifentanil makes this our preferred agent where a continuous opioid infusion is required [23]. The short duration of action of remifentanil allows it to be dosed on lean body mass and this is an advantage. However, its rapid addictive effect, strong hyperalgesia potential and other opioid side effects place limits on remifentanil’s usefulness as a sedative in the ICU. In non-intubated obese patients with a large neck circumference, the risk of inducing or worsening OSAS is a substantial concern.

#### Volatile anaesthetics

Stability of context-sensitive half-time and moderate effects on the respiratory system make volatile anaesthetics an attractive proposition for sedation in the morbidly obese (Table 1) [16]. Individual studies have provided positive comparisons with more conventional sedatives [24]. However, substantial, controlled demonstrations of their effectiveness and safety are absent and volatile anaesthetics are not officially indicated for sedation. Reports on the effects of long-term isoflurane delivered by the CE-approved AnaConDa® device (Sedana Medical, Uppsala, Sweden) [25] illustrate some of the haemodynamic pitfalls that may be encountered with this unapproved use of volatile agents.

Concerns over possible nephrotoxicity with sevoflurane [26,27] and more general anxieties about possible adverse effects of chronic long-term exposure on patients and, more especially, on staff mean that experience is, in our opinion, unlikely to develop beyond the local and anecdotal.

#### Propofol

Surveys of sedation practice on several continents indicate a general tendency against very long-term use of propofol [28-30]. This picture is confounded by observations linking propofol use to reductions in duration of mechanical ventilation and overall ICU length of stay (part of those effects may be due to displacement of long-acting benzodiazepines) [29,31]. In general, however, trends in the use of this drug appear to be consistent with the view that avoiding propofol infusion syndrome is a consideration for many physicians and that for that purpose the duration of a continuous infusion should not exceed 6 days.

Nevertheless, propofol is a well-defined and widely relied upon sedative for short- and medium-term use [32,33]. It has the value and attraction of being familiar to trained anaesthetists even though we regard several of its effects on the respiratory tract as suboptimal in the context of managing obese patients (Table 1). Recently reported experience with fospropofol is also encouraging but in need of further development [34]. Fospropofol is a water-soluble pro-drug and does not deliver the calorie content of traditional propofol emulsion. This may be a consideration.

#### Alpha-2 agonists

Alpha-2 agonists offer a range of theoretically positive features in the sedation of morbidly obese patients (Table 1) but suffer from a lack of relevant pharmacokinetic data and the lack of a critical mass of high-grade clinical data. Nothing in the current dataset suggests that these agents are suitable for achieving deep sedation as monotherapy, though they may be combined with other sedatives for procedural deep sedation. Dexmedetomidine has the attraction of not causing respiratory depression or obstructive breathing during sedation [35,36] and should deliver sufficient sympathetic blockade to confer stable blood pressure and heart rate in many patients. Clonidine has an elimination half-time four times that of dexmedetomidine and a correspondingly longer duration of effect [37]: except in hard-to-specify circumstances where extended effect is desired, this is not likely to be an advantage. The apparent dissociation between effects on bispectral index scores and subject rousability suggest that clonidine is only suitable for light sedation. However, in common with dexmedetomidine [38] and other agents such as ketamine [39], it can be used as an adjunct to reduce dosage requirements for analgo-sedatives and is widely used in Germany, apparently for that purpose [39].

#### Ketamine

Use of ketamine is substantially confined to paediatrics, in part because of concerns about its tolerability profile in adults [40]. Systematic analyses suggest that ketamine may have a place in the analgo-sedation repertoire for adults at least for its opioid-sparing effects [41,42] but, owing to its stimulant action on the sympathetic nervous system, it is difficult to regard it as a first-choice sedative. Its use may also be associated with hallucinations [40]. Combining of ketamine with benzodiazepines may reduce the risk of hallucination though this is not a sufficient reason to consider benzodiazepines when otherwise they might not be.

The risk of serious respiratory adverse events with intravenous ketamine is low [43]. In the context of procedural sedation in the emergency department, use of ketamine has been reported in one survey to be a protective factor for sedation-related airway events [44]. The drug has been assessed in broadly favourable terms in that situation [45].

In general, the newer S-ketamine form has a better tolerability profile than the previously available racemate.

Combinations such as ketofol (ketamine plus propofol) and ketodex (ketamine plus dexmedetomidine) have been reported to offer attractive features for procedural sedation but much of that experience has been accrued in paediatrics [46]. The few published investigations of procedural sedation in adults are encouraging [47,48] but the place of these combinations in the sedation of obese adult ICU patients requires more investigation before conclusions may be reached and recommendations issued. Matters in need of consideration include determination of the optimal dose ratios and structured appraisal of adverse reactions [49].

### The future

Perhaps the most striking aspect of Table 1 is how much current practice in this setting rests on opinion rather than evidence [50].

The to-do list for the immediate future to rectify that situation includes:

- acquiring robust pharmacokinetic data for the alpha-2 agonists and for S-ketamine in obese patients
- controlled comparisons of clonidine and dexmedetomidine
- full characterization of the effects of benzodiazepines on the upper airways and on respiratory drive/muscle function in obese patients
- evaluation of multimodal sedative regimens.

Other themes we should like to see investigated in morbidly obese ICU patients include:

- investigation of the short-term (in-hospital) and longer-term (≥3 months) impact of sedation choices on cognitive dysfunction [51-54]
- the interplay between sedation delivery and the day–night cycle and sleep quality as it relates to OSAS in non-intubated patients.

## Conclusions

We offer the following practical advice on the sedation of morbidly obese patients vulnerable to OSAS and obesity–hypoventilation syndrome.

1. Avoid supine positioning: respiratory mechanics is improved in the sitting (or ‘beach-chair’) position.
2. Endotracheal tubes should be the default airway choice for morbidly obese patients in most sedation cases.
3. Avoid long-acting sedatives-there should be an intention towards rapid emergence of the patient from sedation after any procedure.
4. Avoid drugs with respiratory depressant effects that reduce the breathing frequency and depth.
5. Avoid drugs that cause or exacerbate obstructive breathing in non-intubated patients.
6. Monitor breathing and depth of sedation.
7. We support the view [55] that there are good reasons to favour remifentanil and dexmedetomidine in particular for the facilitation of awake fibre-optic intubation.

## Abbreviations

BMI: Body mass index
ICU: Intensive care unit
OSAS: Obstructive sleep apnoea syndrome
PEEP: Positive end-expiratory pressure
